# High-sensitivity troponin T release profile in off-pump coronary artery bypass grafting patients with normal postoperative course

**DOI:** 10.1186/s12872-018-0893-2

**Published:** 2018-07-31

**Authors:** Wen Ge, Chang Gu, Chao Chen, Wangwang Chen, Zhengqiang Cang, Yuliang Wang, Chennan Shi, Yangyang Zhang

**Affiliations:** 1 Department of Cardiothoracic Surgery, Shuguang Hospital, affiliated to Shanghai University of TCM, Shanghai, 200021 China; 2 Department of Thoracic Surgery, Shanghai Chest Hospital, Shanghai Jiao Tong University, Shanghai, 200030 China; 3 The First Clinical Medical College of Nanjing Medical University, Nanjing, 210029 China; 4 Department of Hygiene Analysis and Detection School of Public Health Nanjing Medical University, Nanjing, 210029 China; 5 Department of Cardiovascular Surgery, East Hospital, Tongji University School of Medicine, 150 Jimo Road, Shanghai, 200120 China; 6 Key Laboratory of Arrhythmias of the Ministry of Education of China, East Hospital, Tongji University School of Medicine, Shanghai, 200120 China

**Keywords:** High-sensitivity troponin T, Off-pump coronary artery bypass grafting, Release profile

## Abstract

**Background:**

The aim of the study was to investigate the high-sensitivity troponin T (hs-TnT) release profile in off-pump coronary artery bypass grafting (OPCABG) patients with normal postoperative course.

**Methods:**

From January 2015 to October 2016, 398 consecutive OPCABG patients who had normal postoperative courses were enrolled. Blood samples for hs-TnT were collected at several time points and the comparisons among different time points grouped by various factors were further analyzed.

**Results:**

There were 317 male and 81 female patients, with a median age of 64. For 66.1% of the patients, peak hs-TnT occurred at the 24th hour after OPCABG, regardless of the groups divided by different factors. In total, the hs-TnT values were much higher in male group (*P* = 0.035), in patients who need 5 or more bypass grafts (*P* = 0.035) and in patients with high-risk EuroSCORE II assessment (*P* = 0.013). However, we failed to find any significant differences between different age groups (*P* = 0.129) or among different coronary heart disease classifications (*P* = 0.191).

**Conclusions:**

The hs-TnT values were affected by various factors and culminated around the first 24 h following OPCABG. It may provide some useful information for future clinical studies of myocardial biomarkers after OPCABG.

## Background

Myocardial cell injury is inevitable after cardiac surgery, leading to the elevation of various cardiac biomarkers. Myocardial infarction (MI), caused by myocardial injury or coronary artery anomaly, is a major cause of disability and death around the world. Perioperative MI may cause adverse events such as reoperation, longer intensive care unit (ICU)/hospital stay or even hospital deaths after cardiac surgery [1–4]. Currently, MI can be defined by many clinical characteristics including elevated values of biomarkers of myocardial ischemia necrosis, myocardial contrast echocardiography and electrocardiography (ECG) results [5].

It has been proved that postoperative serum cardiac troponin T (TnT) level is correlated with increased morbidity and mortality after coronary artery bypass grafting (CABG) [6–8]. Compared with postoperative TnT, preoperative serum TnT does not offer an additional predictive value [9]. Nevertheless, preoperative troponins, with low cut-off value, could provide better predictive value than postoperative values after preoperative percutaneous coronary intervention (PCI) [10]. Nowadays, the new generation hs-TnT could detect minor myocardial injury with higher sensitivity and has been widely used in cardiac surgery.

Postoperative MI has been well recognized and the consensus on MI has been developed for many revisions. Understanding of release profiles after different cardiac procedures helps clinicians perceive perioperative MI and take measures to avoid relevant adverse events [1]. Previous studies have reported the release profile of hs-TnT after cardiac surgery [1, 11]. However, the number of enrolled patients who underwent OPCABG in previous researches was relatively small and the release profile is of significance for CABG patients with normal postoperative courses. Therefore, we undertook an investigation of the release profile following OPCABG in 398 patients who had normal postoperative courses.

## Methods

### Patients

From January 2015 to October 2016, we consecutively enrolled OPCABG patients who had normal postoperative courses. The exclusion criteria were as follows: (1) emergency operation; (2) postoperative MI; (3) postoperative renal failure; (4) severe postoperative complications; (5) perioperative death; (6) the interval between preoperative MI and surgery less than 3 weeks in patients with preoperative MI. According to the third universal definition of myocardial infarction, CABG-related MI can be defined by the high postoperative troponin level (above 10 × 99th percentile upper reference limit (URL)) in patients with normal baseline troponin values within the first 48 h after surgery. Besides, one of the following occurred can also be recognized as CABG related MI: (1) new pathological Q wave or new left bundle branch block; (2) angiographically detected new graft or native coronary artery occlusion; (3) imaging evidence of new regional wall motion abnormality or new loss of viable myocardium [5].

The study was approved by the ethics committees of Shanghai East Hospital (ID2018097). All clinical procedures were performed in accordance with current clinical guidelines and regulations. All patients included in the study, or their legal representatives, signed written informed consents to participate in the study and for all surgical procedures. We reviewed the medical records for all the patients. All the corresponding clinical data including age, sex, body mass index (BMI), coronary artery disease (CAD) classification, New York Heart Association (NYHA) class, comorbidity, preoperative atrial fibrillation, history of PCI, numbers of bypass grafts and the results of serological examination were collected.

Twenty-four hours before surgery, blood samples were obtained for hs-TnT from venous puncture for the first time. Thereafter, blood samples were collected from the end time of surgery at 6, 12, 24, 48, 72, 96 and 120 h postoperatively. Samples were measured in the central laboratory of hospital by standard techniques. Plasma levels of cardiac hs-TnT were measured on Roche CARDIAC reader (Roche cobas E 411). The detection limit is 0 ng/L to 10,000 ng/L. The normal limit is 0 ng/L to 14 ng/L.

### Statistics

All the clinical data were analyzed by using SPSS 19.0 software package (SPSS Inc., Chicago, IL). Variables among patients include gender, age, number of bypass grafts, EuroSCORE II, and CAD classification. If continuous variables conform to the normal distribution, then variables will be expressed as mean ± standard deviation, else variables will be expressed as median and interquartile range (IQR). The distributions of hs-TnT classified by these factors were estimated by using Prism 5.0 (Graph Pad Software Inc., La Jolla, CA) and the last observation carried forward (LOCF) method was used to deal with dropout data of hs-TnT [12]. The difference of hs-TnT at different points was compared by repeated measurements. All *P* values were two-sided and statistical significance was set at 0.05.

## Results

Overall, there were 3184 monitoring points and 99 of them had incomplete hs-TnT data (99/3184, 3.11%). Every patient had their pre-operative data. At 6, 12, 24, 48, 72, 96 and 120 h after surgery, the number of patients who failed in data collection was 14, 14, 16, 17, 9, 14 and 15 respectively. Missing rate at each time point was less than 5%. Three hundred ninety-eight OPCABG patients with normal postoperative course were enrolled in our study. There were 317 male and 81 female, with a median age of 64(11). Of the 398 patients, there were 244 (61.3%) patients with stable angina pectoris, 109 (27.4%) patients with unstable angina pectoris and 45 (11.3%) patients with acute myocardial infarction, respectively. All the 45 patients with acute MI received surgery after 3 weeks. As for NYHA class, patients with class II accounted for the majority (248, 62.3%). Fifteen patients had PCI operations before. Besides, during the operation, the mean numbers of bypass grafts were 3.9 (range from 1 to 6) (Table 1).

**Table 1:** Baseline clinical characteristics of OPCABG patients

Variables	Total (*N* = 398)
Age (y)	^△^64(11)
Female (n, %)	81(20.4)
Weight (kg)	69.03 ± 10.46(37–125)
Height (cm)	166.79 ± 7.18(144–184)
BMI (kg/m^2^)	24.75 ± 2.90(15.60–38.58)
Diabetes (n, %)	106(26.6)
Hypertension (n, %)	218(54.8)
Stroke (n, %)	3(0.8)
Peripheral vascular disease (n, %)	10(2.5)
Previous PCI (n, %)	15(3.8)
Atrial flutter and fibrillation (n, %)	6(1.5)
Pulmonary hypertension (n, %)	108(27.1)
Acute myocardial infarction (n, %)	45(11.3)
Unstable angina pectoris (n, %)	109(27.4)
NYHA class II (n, %)	248(62.3)
LVEF (%)	60.56 ± 8.01(32.7–72.8)
Number of grafts (n)	3.92 ± 1.05(1–6)

^△^Values of variables stand for median and interquartile range (IQR)

Abbreviations: *BMI* body mass index, *COPD* chronic obstructive pulmonary disease, *Scr* Serum creatinine, *Ccr* endogenous creatinine clearance rate, *LVEF* left ventricular ejection fraction

### Hs-TnT profiles

**Fig. 1:**
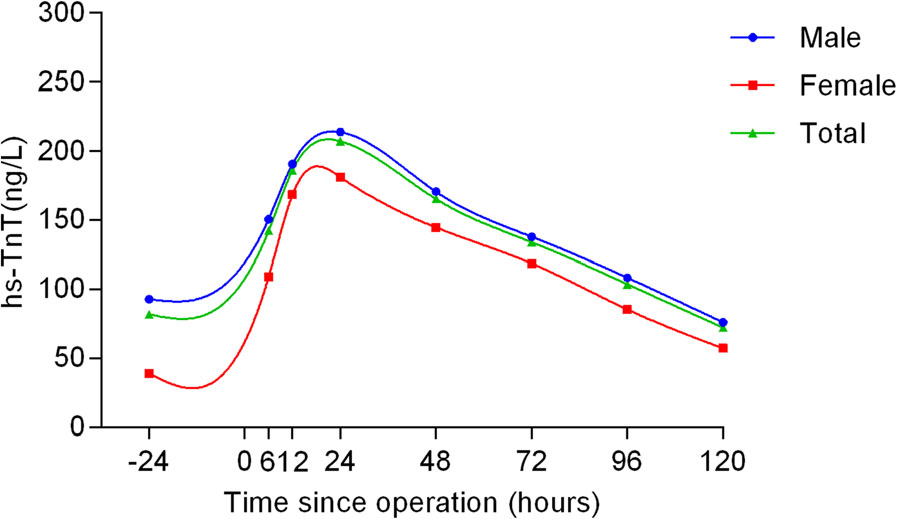
Comparisons among different time points grouped by gender. The blue curve represented male and the red curve represented female, while the total population curve was in green

**Fig. 2:**
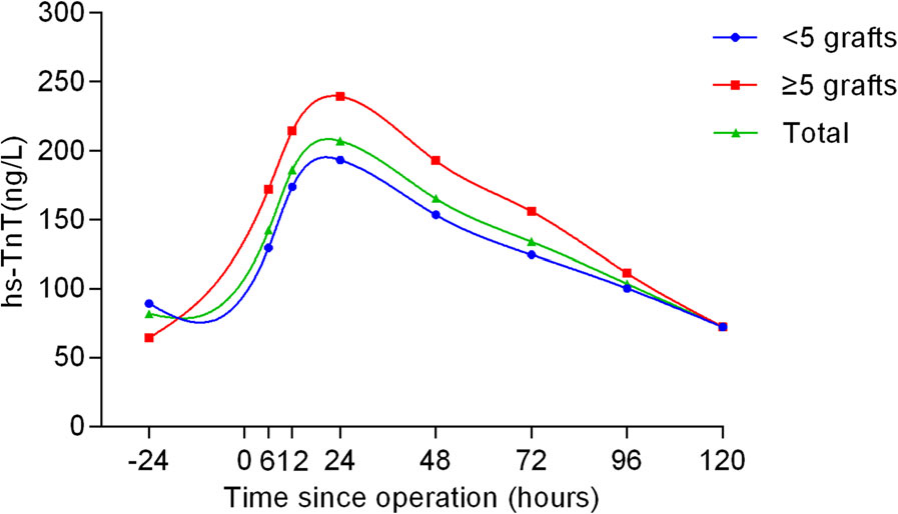
Comparisons among different time points grouped by the number of grafts. The blue curve represented patients who need less than 5 bypass grafts and the red curve represented patients who need 5 or more bypass grafts, while the total population curve was in green

**Fig. 3:**
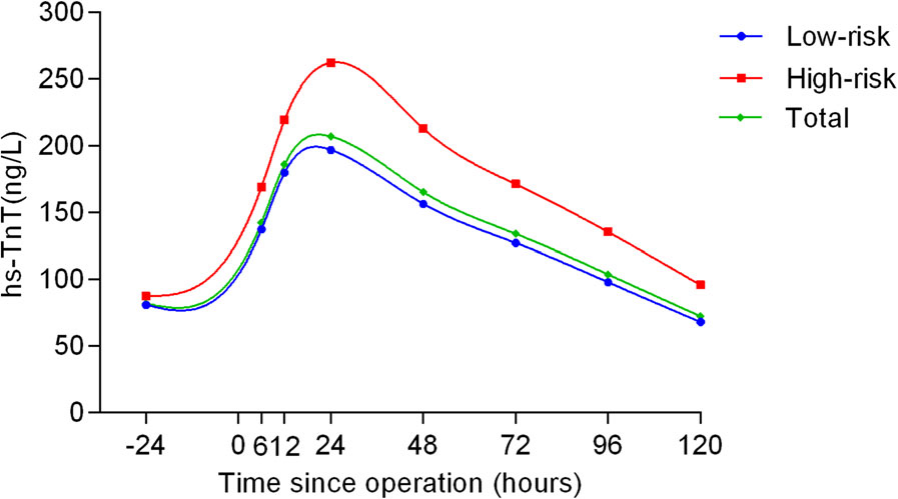
Comparisons among different time points grouped by EuroSCORE. The blue curve represented low risk group (EuroSCORE II, < 2.00) and the red curve represented high-risk group (EuroSCORE II, > = 2.00), while the total population curve was in green

**Fig. 4:**
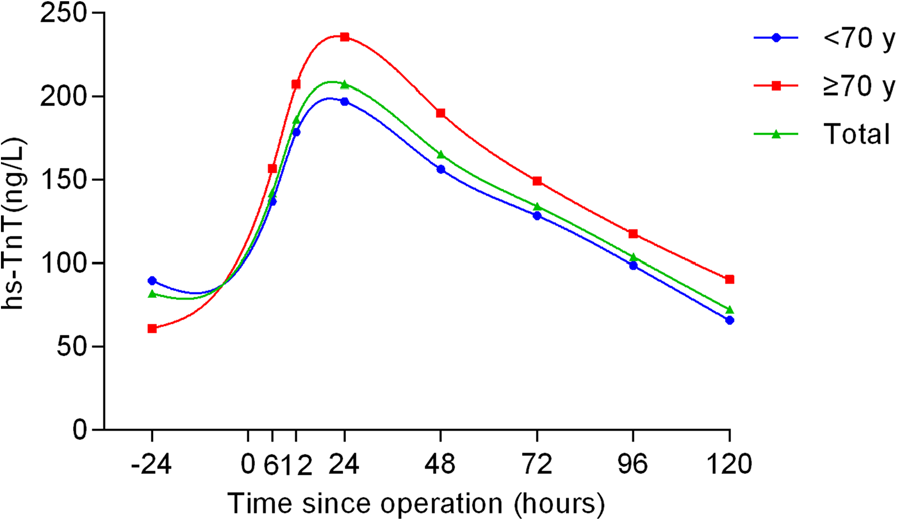
Comparisons among different time points grouped by age. The blue curve represented patients younger than 70 and the red curve represented patients older than 70, while the total population curve was in green

**Fig. 5:**
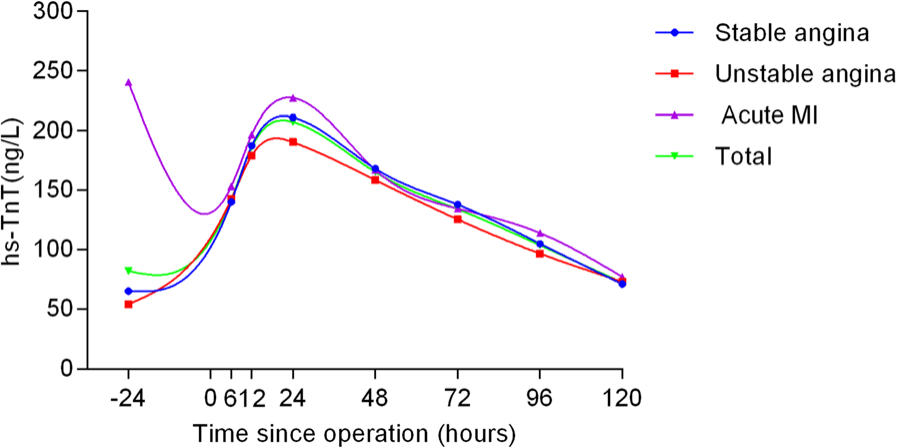
Comparisons among different time points grouped by CAD classifications. The blue curve represented patients with stable angina, the red curve represented patients with unstable angina, the purple curve represented patients with acute MI while the total population curve was in green

The hs-TnT profiles and comparisons are shown in Table 2 and Figs. 1, 2, 3, 4 and 5 according to different factors including gender, age, number of bypass grafts, EuroSCORE II, and CAD classification. Values of TnT stand for means±standard deviation (SD). During perioperative period (24 h before surgery to 120 h after surgery), the profiles all experienced wide variability. Peak hs-TnT value occurred at the 24th hour after OPCABG, regardless of the groups divided by different factors.

**Table 2:** Comparisons among different time points grouped by various factors

	24 h before OP	PO 6 h	PO12h	PO 24 h	PO 48 h	PO 72 h	PO 96 h	PO 120 h
Total	81.98 ± 367.60	142.27 ± 11,208	186.12 ± 145.36	207.13 ± 176.81	165.30 ± 149.34	134.06 ± 123.41	103.74 ± 100.68	72.34 ± 79.52
Group by gender								
Male (*n* = 317)	92.92 ± 407.98	150.75 ± 112.65	190.64 ± 141.43	213.76 ± 176.88	170.57 ± 151.19	137.99 ± 124.17	108.40 ± 106.46	76.19 ± 83.69
Female (*n* = 81)	39.14 ± 103.83	109.08 ± 103.97	168.45 ± 159.53	181.18 ± 175.21	144.65 ± 140.91	118.69 ± 119.91	85.48 ± 71.50	57.30 ± 58.54
Group by grafts								
G1 (*n* = 279)	89.39 ± 424.06	129.51 ± 104.26	173.99 ± 143.33	193.35 ± 169.58	153.56 ± 146.50	124.67 ± 123.86	100.44 ± 103.31	72.31 ± 84.31
G2 (*n* = 119)	64.58 ± 174.74	172.18 ± 123.97	214.18 ± 146.72	239.45 ± 189.54	192.82 ± 152.92	156.07 ± 120.01	111.48 ± 94.21	72.44 ± 67.28
Group by EuroSCORE II								
HRG (*n* = 62)	87.46 ± 176.87^*^	169.09 ± 125.95^**^	219.53 ± 169.83^***^	262.19 ± 275.68^****^	213.12 ± 230.94^*****^	171.18 ± 191.37^******^	135.39 ± 148.29^*******^	95.81 ± 113.85^********^
LRG (*n* = 336)	80.96 ± 392.99	137.32 ± 108.81	179.96 ± 140.89	196.97 ± 150.14	156.47 ± 127.35	127.19 ± 105.25	97.90 ± 88.26	68.01 ± 70.80
Group by age								
Elderly (*n* = 105)	89.60 ± 420.82	137.04 ± 105.12	178.56 ± 138.02	196.92 ± 149.48	156.27 ± 124.11^∆^	128.52 ± 106.91	98.69 ± 88.02	65.83 ± 62.79 ^∆∆^
Young (*n* = 293)	60.97 ± 139.73	156.66 ± 128.78	206.96 ± 162.79	235.27 ± 234.91	190.16 ± 201.97	149.33 ± 159.97	117.65 ± 128.80	90.18 ± 111.98
Group by CAD								
SAP (*n* = 244)	65.21 ± 368.34^▲^	140.13 ± 97.59	187.26 ± 145.49	187.26 ± 145.49	210.96 ± 192.52	168.01 ± 163.61	104.99 ± 105.93	71.13 ± 80.48
USAP (*n* = 109)	54.09 ± 142.57	142.55 ± 131.44	179.22 ± 135.21	190.28 ± 134.15	158.48 ± 113.69	125.45 ± 87.68	96.70 ± 72.59	73.07 ± 69.93
AMI (*n* = 45)	240.38 ± 625.21	153.19 ± 134.88	196.69 ± 169.23	227.18 ± 178.92	167.10 ± 146.69	134.42 ± 113.17	113.97 ± 127.76	77.16 ± 96.16

Abbreviations:*G*1 group1, number grafts ≤4; *G*2 group2, number grafts ≥5, *HRG* high risk group (EuroSCORE II > =2.00%), *LRG* low risk group (EuroSCORE II < 2.00%), *CAD* coronary atherosclerotic heart disease, *SAP* Stable angina pectoris, *USAP* Unstable angina pectoris, *AMI* acute myocardial infarction, *OP* operation, *PO* postoperative

^*^P = 0.003; ^**^ *P* = 0.040; ^***^ *P* = 0.049; ^****^ *P* = 0.007; ^*****^ *P* = 0.006; ^******^ *P* = 0.010; ^*******^ *P* = 0.007; ^********^ *P* = 0.011; ^∆^ *P* = 0.045; ^∆∆^ *P* = 0.007; ^▲^ *P* = 0.008

When grouped by gender, the hs-TnT values were much higher in male group in total (*P* = 0.035). Besides, when compared at each time point, the values were all higher in men, and only at the 6th hour after surgery did the hs-TnT value have statistical discrepancy (*P* = 0.003) (Fig. 1).

When grouped by number of bypass grafts, the hs-TnT values were much higher in patients who need 5 or more bypass grafts (*P* = 0.035). In addition, when compared at each time point, the hs-TnT values in patients with 5 or more bypass grafts were much higher at 6 (*P* < 0.001), 12 (*P* = 0.011), 24 (*P* = 0.017), 48 (*P* = 0.016) and 72 (*P* = 0.020) hours after surgery, respectively (Fig. 2).

When grouped by EuroSCORE II, there was statistical difference between high-risk group (EuroSCORE II,> = 2.00) and low risk group (EuroSCORE II,< 2.00) (*P* = 0.013). Furthermore, when compared at each time point, the hs-TnT values were all higher in high-risk group and all had significant differences after surgery (Fig. 3).

When grouped by age, in total, although the hs-TnT values in elderly patients (> = 70 y) were higher, we failed to find any significant differences between the two groups (*P* = 0.129). However, when compared at each time point, there were significant differences at 48 (*P* = 0.045) and 120 (*P* = 0.007) hours after the surgery (Fig. 4).

When grouped by CAD classification, this factor did not have significant impact on the variability of hs-TnT values (*P* = 0.191). Interestingly, when compared at each time point, only preoperative values had significant differences (*P* = 0.008) (Fig. 5).

## Discussion

Elevation of cardiac biomarkers was a common phenomenon following CABG, which was ascribed to a number of causes including, though not limited to, cardiomyocyte death from insufficient myocardial protection, embolism, regional or/and global ischemia and surgical procedure injuries. Of all the covariates predictive of mortality, the elevation of troponins in the first 24 h following CABG is one of the strongest correlative factors [13]. Therefore, it is worthy to identify postoperative MI and differentiate expected elevation of troponins from unexpected suspicious perioperative MI in order to guide clinical decisions. Carmona et al. [14] indicated that both CABG and OPCABG are safe and there were no statistically significant differences in terms of long-term all-cause mortality. However, they found OPCABG was related with less postoperative morbidity and shorter ICU and hospital stay. Currently, there is no consensus on hs-TnT release profile after OPCABG. When compared with conventional TnT, hs-TnT has higher sensitivity especially when measured early after symptom onset and has the advantage in early diagnosis of acute MI [15–17]. The present study showed that, in OPCABG patients with normal postoperative course, the hs-TnT could sensitively reflect the extent of myocardial damage and its value peaks around the 24th hour after surgery.

With normal postoperative course, the release of hs-TnT differed among individuals undergoing OPCABG. Various factors contributed to the variation in hs-TnT release. The serum hs-TnT level can be influenced by patient factors and perioperative factors. Gore et al. [18] demonstrated that the uniform 14 ng/l cut-off value for hs-TnT may lead to over-diagnosis of perioperative MI in male or elderly patients because men and the elderly have higher normal URL. In our series, although high level (more than 10 times of URL) of hs-TnT has been found in some patients, they did not have concomitant symptoms like (1) new pathological Q wave; (2) angiographically detected new graft or native coronary artery occlusion (3) imaging evidence of new regional wall motion abnormality or new loss of viable myocardium [5]. Therefore, they could not be defined as postoperative MI, but could suggest a certain degree of myocardial damage. Besides, some comorbidities like hypertension, diabetes mellitus, ventricular hypertrophy and the efficacy of plasma troponin clearance can also affect hs-TnT level [1, 19, 20]. Perioperative factors like cardiotonic drug usage and cardiac arrhythmia are proved to have an impact on the hs-TnT values [21].

In our series, there was an interesting phenomenon that values of postoperative hs-TnT were much greater than URL. Jorgensen et al. reported that they observed 99 patients undergoing CABG and the cardiac troponin (cTnI) levels were far above the cut-off point of guideline. The median peak value in normal postoperative course was 7675 ng/L, which was 255 times the URL [22]. Another literature also reported the same results [23–25]. The possible explanation for this phenomenon was that the complexity and multi-factors of cardiac surgery led to myocardial damage, which was more serious than PCI.

As for patient factors, increased age and impaired renal function contributed to high level of serum hs-TnT, and thus affecting diagnostic accuracy for acute MI [26]. Elderly age (> 70 years) has been considered as a significant risk factor for elevated hs-TnT. As previous study reported, regardless of the final diagnosis, hs-TnT concentrations in elderly patients were much higher than that in younger patients (20.9 ng/L versus 3.9 ng/L) [26]. These results were basically consistent with ours. In our study, the hs-TnT values in elderly patients were higher. Furthermore, when compared at each time point, there were significant differences at 48 (*P* = 0.045) and 120 (*P* = 0.007) hours after the surgery. The reason would be that when renal function declined from age, elderly patients will have low estimated glomerular filtration rate. As a result, hs-TnT could not be eliminated in time, leading to relatively high hs-TnT level in peripheral blood.

Among patients with different CAD types, there were significant differences when comparing the data of the 24th hour before operation, which were in accordance with patients’ condition. Postoperatively, there were no statistical differences among them and the results indirectly reflected the effectiveness of surgery. As for EuroSCORE II, the score itself has included comprehensive assessments of patients. High-risk patients had more risk factors than low-risk ones. The chances for myocardial damage increase. As a result, the hs-TnT level was higher in high-risk group.

There are some limitations in our study. First, although this study contains 398 OPCABG patients, the sample size is still relatively small. Second, this is a retrospective designed study, which may cause a certain degree of selection bias. Third, there would be interaction among different perioperative factors, which may affect hs-TnT level, leading to the exclusion of patients with false positive perioperative MI.

## Conclusions

Our study demonstrated that the hs-TnT release profile in OPCABG patients who had normal postoperative courses and the hs-TnT values were affected by various factors and often peak around the 24th hour following OPCABG. It may provide some useful information for future clinical studies of myocardial biomarkers after OPCABG.

## Abbreviations

BMI: Body mass index
CABG: Coronary artery bypass grafting
CAD: Coronary artery disease
cTnI: Cardiac troponin
ECG: Electrocardiography
hs-TnT: High-sensitivity troponin T
ICU: Intensive care unit
IQR: Interquartile range
LOCF: Last observation carried forward
MI: Myocardial infarction
NYHA: New York Heart Association
OPCABG: Off-pump coronary artery bypass grafting
PCI: Percutaneous coronary intervention
SD: means±standard deviation
TnT: Troponin T
URL: Upper reference limit
